# DEMENTIA AND MILD COGNITIVE IMPAIRMENT: PREVALENCE, CONVERSION RATES, AND LONGEVITY BETWEEN AGES 70 AND 95

**DOI:** 10.1093/geroni/igad104.2405

**Published:** 2023-12-21

**Authors:** Jochanan Stessman, Jeremy Jacobs

**Affiliations:** Hebrew University of Jerusalem, and Hadassah Medical Center, Israel, Jerusalem, Yerushalayim, Israel; Faculty of Medicine, Hebrew University of Jerusalem, and Hadassah Medical Center, Israel, Jerusalem, Yerushalayim, Israel

## Abstract

This study describes the prevalence and conversion rates of Mild Cognitive Impairment (MCI) and dementia between age 70-95, and the association between cognitive status and mortality. The Jerusalem Longitudinal Study (1990-2023) prospectively follows a representative community-dwelling cohort born 1920-21, assessed at home visit during 1990,1998, 2005, 2010, 2015 at ages 70, 78, 85, 90, and 95 (n=437, 806, 1170, 584, 468) respectively. Comprehensive assessment included Mini Mental State Examination (MMSE), defining normal cognition (29-30/30), MCI (24-28/30) and dementia (≤23/30). Mortality data were collected from the Ministry of Interior. Cox proportional hazards ratios (HR) were determined, adjusting for gender, education, self-rated health, physical activity, BADL status, diabetes, hypertension, ischemic heart disease. At ages 70, 78, 85, 85, 90, and 95 the prevalence of MCI was 13.5%, 21.5%, 38.7%, 37.0%, and 50%; prevalence of dementia was 3.2%, 4.2%, 24.3%, 31.3%, 35.7%. Between age 70-78, 78-85, 85-90, 90-95, conversion rates from MCI back to normal were 74.2%, 27.4%, 18.2%, 7.3%; MCI declining to dementia were 6.5%, 29.2%, 37.0%, 50.9%; MCI remaining MCI were 19.4%, 43.4%, 44.8%, 41.8%. Dementia was significantly associated with increased mortality throughout the entire study period: at age 70-78 Hazards Ratio=4.36 (CI 95%: 2.09-9.10), age 90-95 HR=1.5 (CI 95%: 1.01-2.21). In contrast, MCI was not associated with increased mortality, apart from at age 85: HR=1.38 (CI 95%: 1.04-1.82). Our findings show that conversion rates from MCI to dementia are age-dependent, with less than half of MCI subjects progressing to dementia. Unlike dementia, MCI was largely unassociated with mortality.

